# Computational Thermomechanical Properties of Silica–Epoxy Nanocomposites by Molecular Dynamic Simulation

**DOI:** 10.3390/polym9090430

**Published:** 2017-09-08

**Authors:** Xiaoxing Zhang, Hao Wen, Yunjian Wu

**Affiliations:** School of Electrical Engineering, Wuhan University, Wuhan 430072, China; wenhao198711@163.com (H.W.); wuyunjian@whu.edu.cn (Y.W.)

**Keywords:** silica–epoxy, glass transition temperature, grafting ratio, thermal conductivity, mechanical properties

## Abstract

Silica–epoxy nanocomposite models were established to investigate the influence of silane coupling agent on the structure and thermomechanical properties of the nanocomposites through molecular dynamics simulation. Results revealed that incorporating silica nanoparticles into a polymer matrix could improve thermomechanical properties of the composites and increase their glass transition temperature and thermal conductivity. Their thermomechanical properties were further enhanced through silane coupling agent modification on the surface of fillers. Compared with that of pure epoxy, the glass transition temperatures of the silica–epoxy composites with grafting ratios of 5% and 10% increased by 17 and 28 K, respectively. The thermal conductivities of the two models at room temperature respectively increased by 60.0% and 67.1%. At higher temperature 450 K, thermal conductivity of the nanocomposite model with a high grafting ratio of 10% demonstrated a considerable increase of approximately 50% over the pure epoxy resin (EP) model. The elastic and shear modulus of the nanocomposite models decreased at temperatures below their glass transition temperatures. These observations were further addressed in the interpretation from three aspects: segmental mobility capability, radial distribution function, and free volume fraction. Our computational results are largely consistent with existing experimental data, and our simulation model got fully validated.

## 1. Introduction

Epoxy resin (EP) has been widely used in power transmission and transformation equipment because of its numerous advantages, such as good adhesiveness, chemical stability, good mechanical properties, flexible processing, low shrinkage, and low cost. With the construction of high-voltage power grids, electrical equipment has been developed in terms of environmentally friendliness, high efficiency, and smaller occupied area. Hence, new dielectric materials with an excellent insulation performance, good aging resistance and adequate mechanical properties have been proposed [1]. However, common thermosetting epoxy resins fail to satisfy all of these standards, and high breakdown strength may not guarantee high thermal and mechanical properties. To overcome this problem, researchers prepared nanocomposites based on epoxy resin hosts [2]. Incorporating nanoparticles into a polymer host can improve the thermal properties of composites, enhance aging resistance, and maintain supportive strength and toughness [3,4,5,6].

Mathematic models and evaluation methodology have been established to assess the comprehensive properties of nanocomposites for practical applications. Electrical, thermal, and mechanical properties can be enhanced through incorporating nanoparticles into polymer hosts, and their effects have been elucidated on the basis of multiple perspectives, such as particle size, shape, amount, and interface interaction characteristics; among these parameters, size and interface interaction possibly play a predominant role [7,8,9,10,11,12,13,14,15]. Some principle models have also been created to illustrate the connection mechanism and depict the reactions of particles with polymer hosts. However, the effects of size, shape, surface modification, and interface interaction have been poorly explained or inevitably neglected under various conditions, especially when the size of particles reach a nanometer scale [16,17,18]. With the rapid development of molecular simulation technology, molecular dynamics simulation has shown advantages for research on the microstructures of nanocomposites and therefore has served as an ideal approach to predict relative physical properties. Given that molecular computational science depending on energy calculation can provide strong evidence to verify previous experimental results and determine preliminary properties, researchers design new dielectrics by using this practical approach to fabricate desirable products, avoid potential material wastage, and reduce difficulties in conducting property tests [19,20,21,22,23].

Epoxy resin nanocomposites have been widely simulated through molecular dynamics. A series of thermal and mechanical parameters, such as volumetric shrinkage, glass transition temperature (*T*g), coefficient of thermal expansion (CTE), elastic modulus, and Poisson’s ratio as a function of different crosslinking degrees, is calculated through molecular dynamics simulations, and computational results are largely consistent with existing principle models [24]. Bandyopadhyay also conducted similar studies by using a new EPON 862–DETDA epoxy system (Wuhan Shen Chemical Reagents and Equipments Co., Ltd., Wuhan, China) [25]. Choi proposed an effective approach to evaluate the influence of different particle sizes on *T*g and CTE and obtained results that match well with those from a finite element method [26]. Jeyranpour investigated the effects of temperature and doping amount on the thermomechanical properties of EP/fullerene nanocomposites and observed that *T*g decreases with temperature, whereas the mechanical properties increase with fullerene doping amount [27]. The thermal properties of EP/graphene and EP/SWNT nanocomposites have also been fully quantified in terms of different sizes, layers, length-to-diameter ratios, and arrangement types. Adding the two specific fillers grapheme and SWNT with high thermal conductivities will help improve the thermal properties of nanocomposites [28,29].

Silica, as a common filler of epoxy-hosted dielectric composites, has been rarely examined through computational approaches, although experimental studies have indicated that silica can highly enhance the thermomechanical properties of composites [30,31]. Silica–epoxy composites have been simulated through molecular dynamics to introduce the influence of crosslinking density and particle surface treatment, but a systematic or detailed interpretation of structure–property relationship has yet to be presented [32,33]. In our study, advanced molecular dynamics simulations were applied to quantitatively predict various properties, including *T*g, CTE, thermal conductivity, and elastic and shear modulus of a silica–epoxy system in the presence of a silane coupling agent. Another three analysis methods, namely, segmental mobility, radial distribution function (RDF), and free volume fraction calculation, were adopted to explain the predicted data. Finally, simulation models were then validated.

## 2. Computational Methods

### 2.1. Establishement of Models

**Figure 1:**
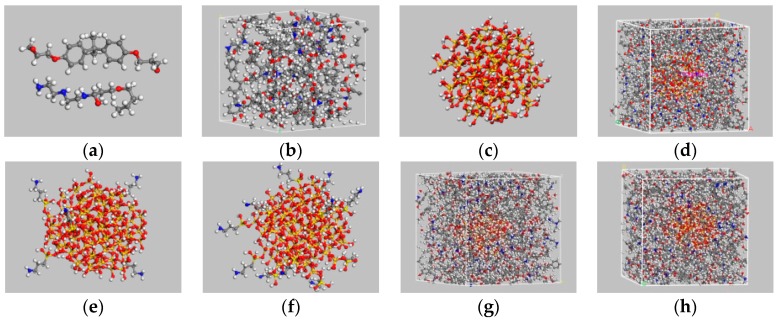
The molecular structure and unit cells of the established models. (**a**) chemical structures of DGEBA and one curing agent; (**b**) structure of pure EP model after curing; (**c**) spherical structure of one SiO_2_ particle with a diameter of 10 nm; (**d**) 3D crosslinking structure of SiO_2_/EP; (**e**) structure of one SiO_2_ particle with surface modification at a grafting ratio of 5%; (**f**) structure of one SiO_2_ particle with surface modification at a grafting ratio of 10%; (**g**) 3D crosslinking structure of SiO_2_–5%/EP; (**h**) 3D crosslinking structure of SiO_2_–10%/EP.

**Figure 2:**
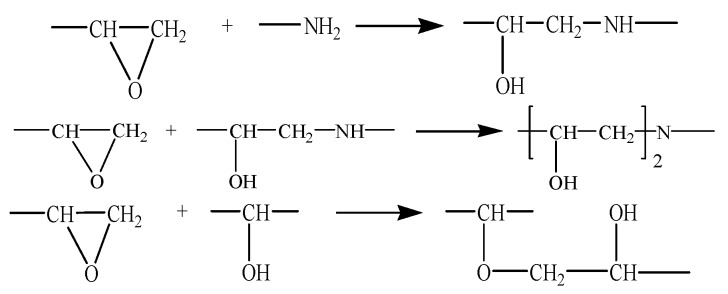
Three main chemical reactions during the curing process.

Materials Studio software packages (Accelrys Co., Ltd, San Diego, CA, USA)were used for the molecular dynamics simulations performed in this study. Four kinds of periodic unit cells were prepared to compare the surface of silica particles before and after they were modified using a silane coupling agent. The four unit cells were described as follows: (1) pure epoxy unit cell, (2) silica–epoxy composite unit cell (SiO_2_/EP), (3) silica–epoxy composite unit cell with 5% grafting ratio of silane coupling agent (SiO_2_–5%/EP); and (4) silica–epoxy composite unit cell with 10% grafting ratio of silane coupling agent (SiO_2_–10%/EP). Bisphenol A epoxy resin (DGEBA) was selected as a polymer host, and an aliphatic amine curing agent named 593 is composed of adducts of diethylene triamine and butyl glycidyl ether. The average diameter of the spherical silica particles was 10 nm. The following molecular modeling was conducted:

1. Cell construction: single molecular models of DGEBA and the curing agent were manually designed (Figure 1a), and the polymerization degree of DGEBA molecules was maintained at a fixed value of 0 to simplify the crosslinking process. A periodic 3D structure consisting of 16:8 molecular mixture of DGEBA and cross linker monomers was then established. The initial density of the unit cell was presumed 0.6 g/cm^3^.
2. For pre-equilibration, a slow stress relaxation procedure was performed to obtain an equilibrated structure. The geometric optimization of 5000 iterations was conducted to acquire a slightly deformed unit cell with a lower total energy than the initial value. Molecular dynamics simulations were subsequently conducted in an NVT (constant volume and temperature) ensemble for 100 ps at 298 K. In the NVT ensemble, forcefield COMPASS was selected and Andersen was used to control temperature. Afterwards, the unit cell was equilibrated twice under the NPT ensemble at 100 ps and 1 atm to obtain the final density of above 1 g/cm^3^, and a time step of 1 fs should be used. In an NPT (constant pressure and temperature) ensemble, Andersen and Berendsen were chosen to regulate temperature and pressure, respectively. The last equilibrated unit cell was utilized for the subsequent crosslinking step.
3. Three main chemical reactions were involved in the formation of 3D cross-linked thermoset epoxy (Figure 2). Before ring-opening reaction occurs, active –NH·group and –CH(OH)–CH_2_ group were manually prepared. Then, 16 DGEBA molecules and eight curing agent molecules with active reaction groups formed periodic unit cells and underwent a series of new bond formation processes under the following controlled parameters: initial cutoff distance of 3.5 Å, maximum cutoff distance of 7.0 Å, and conversion degree of 85%. After a specific crosslink PERL language was run, the final model was obtained (Figure 1b). Each final cross-linked structure was equilibrated over 2000 ps NPT dynamics at 298 K and 1 atm to reach the most stable structure before computing thermal and mechanical properties.
4. A spherical SiO_2_ particle with a diameter of 10 Å was established in accordance with a previously described method [33,34]. Hydroxylation was conducted to produce Si–OH on the surface of the SiO_2_ particle and consequently achieve a good match with experimental results. Then, one unit cell of a silica–epoxy composite could be established at a controlled density of 0.6 g/cm^3^. A cross-linked silica–epoxy composite unit cell could be obtained after steps 2 and 3 were repeated (Figure 1d).
5. Manual grafting was conducted to remove a fixed number of hydrogen atoms from the total hydroxyl groups and graft some KH550 molecules as the silane coupling agent to connect to SiO_2_ particles through Si–O bonds [35]. Grafting involved a series of hydrolysis and coupling reactions. Figure 1c shows the particles with a diameter of 10 nm and 104 hydroxyls on the surface. Five and ten KH550 molecules were respectively added onto the surface to form two kinds of SiO_2_ models with 5% and 10% grafting ratios (Figure 1e,f). The reactions between –NH_2_ group at the end of KH550 molecules and epoxy groups could be disregarded because of the low active energy of –NH_2_ groups. Figure 1g,h show the final crosslinking 3D structure of nanocomposites models with 5% and 10% grafting ratios, respectively.

### 2.2. Tg and CTE

*T*g and CTE are considered to analyze the thermal property of composites because the density and volume of materials linearly or nonlinearly vary with temperature. *T*g of epoxy resin is determined according to the relationship curve between specific volume (the reciprocal of density) and temperature. The inflection point can be obtained through the linear fitting of specific volume as a function of temperature, below and above the approximate *T*g range, and the corresponding temperature of the intersection point is *T*g of the epoxy crosslinking system [36]. CTE refers to a material’s dilation attributed to changes in temperature, and this parameter can be used to measure residual stress formed during polymer processing. CTE is calculated using Equation (1):

(1) $$\alpha =\frac{1}{V_{0}}{\left(\frac{\partial V}{\partial T}\right)}_{p},$$

where *V*_0_ and *P* are the initial volume and pressure of the system, respectively. The linear fitting curve of the specific volume to temperature indicates that the slopes of two straight lines below and above *T*g directly represent CTE. The specific volumes of polymers and polymer-based composites change rapidly when their temperature increase as they pass through the glass transition region. Thus, polymeric materials have two different CTEs, one for the glass state (250–400 K) and the other for the rubbery state (425–650 K).

The established molecular models were used as the initial configuration of our system. After the equilibration at 298 K, the temperature of each unit cell of four kinds of models was increased to 650 K, and the cells were equilibrated again at that temperature at 1 atm for 200 ps. Then, a gradual cooling-down simulation was implemented at a constant cooling rate of 25 K/250 ps until the temperature of each unit cell reached 250 K. The details are as follows:

The models were initially equilibrated by an isothermal and isochoric (NVT ensemble) molecular dynamics simulation at 50 ps and 600 K and then by an isothermal and isobaric (NPT ensemble) molecular dynamics simulation at 200 ps and atmospheric pressure. These models were subsequently cooled at a rate of 25 K/250 ps from 650 to 250 K in nine stages, that is, 50 ps—long NVT simulations were performed for each model and 200 ps long NPT simulations were carried out at all temperatures. Thus, an NPT simulation was additionally applied to smaller systems for 200 ps after the cooling-down simulation finished. Finally, the specific volume—temperature plots were obtained after the curve was linearly fitted.

To investigate the effects of grafting modification of particles on the thermal and mechanical properties, the diameter of nanoparticles was fixed 10 Å and volume fraction was 7.2 vol % for nanocomposites models. Table 1 and Table 2 show the temperature dependence of sizes of cubic unit cells and densities in the cooling-down process simulation, respectively.

Side length of unit cell for pure epoxy resin model was about 30 Å and slightly increased with temperature, whereas, the side lengths of cubic unit cells for the other three nanocomposites models varied in the range of 39 to 41 Å, reflecting the stability of energy. Correspondingly, densities of four models have good agreement with computational and experimental results [33,37].

### 2.3. Thermal Conductivity

Thermal conductivity is an important parameter to represent the thermophysical property of EP materials. This parameter should be improved to enhance the heat dissipation capability of EP materials in order to maintain a relatively lower operating temperature of equipment.

Thermal conductivity has been investigated in relation to molecules or atoms. On the basis of the analog computation of molecular dynamics, we can summarize thermal conductivity investigations as equilibrium molecular dynamics (EMD) and nonequilibrium molecular dynamics (NEMD) methods referring to equilibrium and nonequilibrium states, respectively [38]. According to linear response theory, the EMD method provides traditional approaches, such as Green-Kubo Equation (2) to calculate thermal conductivity:

(2) $$\lambda =\frac{1}{3Vk_{B}T^{2}}\int_{0}^{\infty }\langle J(t)J(0)\rangle dt$$

where *V* is the total volume of the system, k_B_ is the Boltzmann constant, *T* is the temperature unit, and *J* is the microscopic heat flux of the system. However, this method is characterized by several disadvantages, such as slow computation speed and poor convergence. As such, an innovative NEMD method has been introduced to solve thermal conductivity through Fourier’s law under the condition of a fixed temperature difference and heat flux.

RNEMD aims to determine heat flux by exchanging the velocity vector of atoms inside a system. In this study, the thermal conductivity of EP composite models could be simulated through this method because the total energy and momentum of the system remained constant.

**Figure 3:**
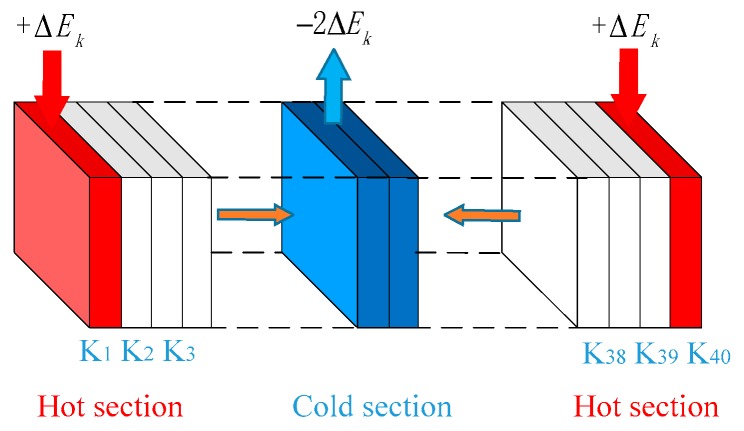
Schematic diagram of RNEMD model.

Figure 3 illustrates the schematic of RNEMD simulation. All of the models were equally divided into 40 parts along the z-axis. Both ends of the model are high-temperature layers while the middle part is a low-temperature layer. An exchange in kinetic energy occurs between the atoms at both ends and those in the low–temperature zone during heat flux spreading toward the middle layers [39,40].

### 2.4. Mechanical Response

Mechanical properties can be determined by calculating small deformations at different static pressures in given directions. The planes of *xy*, *xz*, and *yz* are respectively subjected to small shear deformation along the *x*-, *y*-, and *z*-axis directions to produce 12 groups of strains with the extent of strain maintained within 0.01 in accordance with Hooke’s law, as shown in Equation (3):

(3) $$\sigma_{i}=C_{ij}\varepsilon_{j}.$$

Within the error range, EP crosslinking can be simulated and calculated as an isotropic material. Therefore, stiffness matrix is simplified as follows:

(4) $$\left[\begin{array}{cccccc} \lambda +2\mu & \lambda & \lambda & 0 & 0 & 0\\ \lambda & \lambda +2\mu & \lambda & 0 & 0 & 0\\ \lambda & \lambda & \lambda +2\mu & 0 & 0 & 0\\ 0 & 0 & 0 & \mu & 0 & 0\\ 0 & 0 & 0 & 0 & \mu & 0\\ 0 & 0 & 0 & 0 & 0 & \mu \end{array}\right],$$

where λ and μ are Lamé constants. The mechanical properties, such as elastic modulus (*E*), shear modulus (*G*), bulk modulus (*K*), and Poisson’s ratio (*υ*), of the EP composite system can be expressed as follows:

(5) $$E=\mu \cdot \frac{3\lambda +2\mu }{\lambda +\mu },$$

(6) G=μ,

(7) $$K=\lambda +\frac{2}{3}\mu ,$$

(8) $$\upsilon =\frac{\lambda }{2(\lambda +\mu )}.$$

## 3. Results and Discussion

### 3.1. Tg and CTE

**Figure 4:**
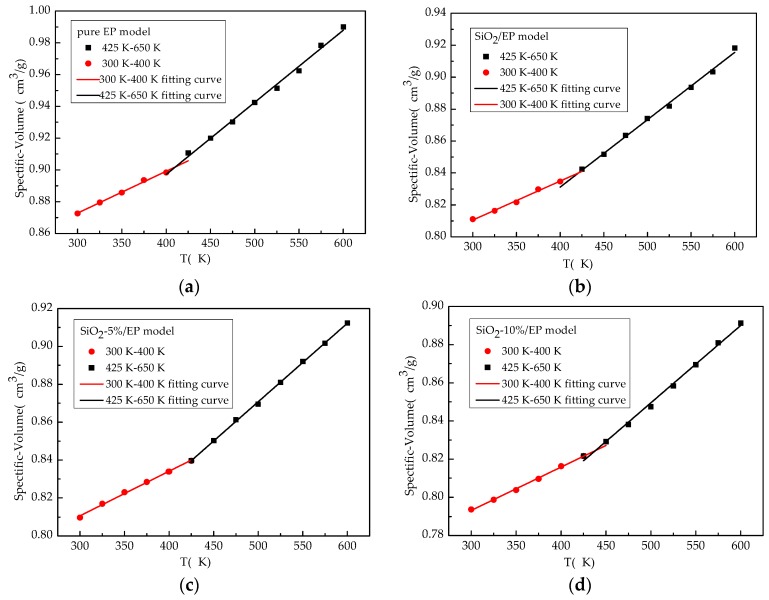
Specific volume curves versus temperature of four models. (**a**) pure EP model; (**b**) SiO_2_/EP model; (**c**) SiO_2_–5%/EP model; and (**d**) SiO_2_–10%/EP model.

Achieving a complete equilibrium state is difficult because of a large number of atoms in a molecular system. Therefore, molecular dynamics calculation can be applied to reach a balanced state when the density of a particular system fluctuates in a small range around the target temperature. In general, 200 ps is set to obtain a well-equilibrated system after NPT simulation is performed. Figure 4 shows the specific volume–temperature plots of the four kinds of models. *T*g was marked as the corresponding temperature at the inflection point of the slopes of specific volume–temperature linear curves. *T*g and the calculated CTE of the four models are summarized in Table 3.

An increasing tendency of *T*g was evident when SiO_2_ particles were added to the epoxy host, especially when *T*g of SiO_2_–epoxy model with 10% grafting ratio of silane coupling agent increased by 7% compared with the pure epoxy resin model. *T*g of SiO_2_/EP composite matched well with experimental data reporting *T*g of 141.2 °C (414.35 K) in Ref. [3]. An adequate bias from the simulated value could be maintained within the allowable range of error because of existing defects during the curing process. As the grafting rate increased, *T*g also increased possibly because of the high conversion degree. By contrast, CTE declined below and above *T*g of the silica–epoxy composite model. Surface modification with the silane coupling agent could obviously reduce CTE and could directly demonstrate the formation of a tight 3D cross-linked structure with a small freedom space in the system.

To further interpret the existing difference among the computational results, the Willliams–Landel–Ferry (WLF) equation can be used to quantitatively account for the dependence of *T*g on the cooling rate as shown in Equation (9) [42]. The WLF equation can relate the difference in *T*g values from simulation and experiment (Δ*T*g) to the relative cooling rate:

(9) $$\Delta Tg=\frac{-C_{2}\log_{10}\frac{{\dot{q}}_{exp}}{{\dot{q}}_{sim}}}{C_{1}+\log_{10}\frac{{\dot{q}}_{exp}}{{\dot{q}}_{sim}}}.$$

In Equation (9), ${\dot{q}}_{exp}$ and ${\dot{q}}_{sim}$ are the experimental and simulation cooling rates, respectively, and $C_{1}$ and $C_{2}$ are the WLF equation parameters. Using the conventional value for WLF parameters ($C_{1}=17.44$ and $C_{2}=51.6\;K$), the predicted shift in the *T*g between experimental measurements (a range of ${\dot{q}}_{exp}$ = 4.88–6.77 K/s [43]) and our simulation (${\dot{q}}_{sim}=100\times 10^{9}\;K/s$) is 72.17–74.63 K, a reasonable agreement between the shifted *T*g from simulation value range of (410–460 K) and experimental value range of (363–420 K) [43].

### 3.2. Thermal Properties

The common mechanical and electrical properties of epoxy resin must be maintained during long-term operations. However, the temperature of devices with epoxy resin materials inevitably increases. As such, a feasible design should be proposed to produce dielectrics with enhanced thermal properties. Usually, the temperature of epoxy resin materials must be kept below 450 K during long-term operations. Therefore, the thermal conductivity of EP composites between 250 and 450 K were investigated through molecular dynamic simulation. Table 4 shows the calculated results for the four models at 250, 300, 350, 400, and 450 K. Five simulations were carried out at each temperature to obtain an average value to reduce errors.

**Figure 5:**
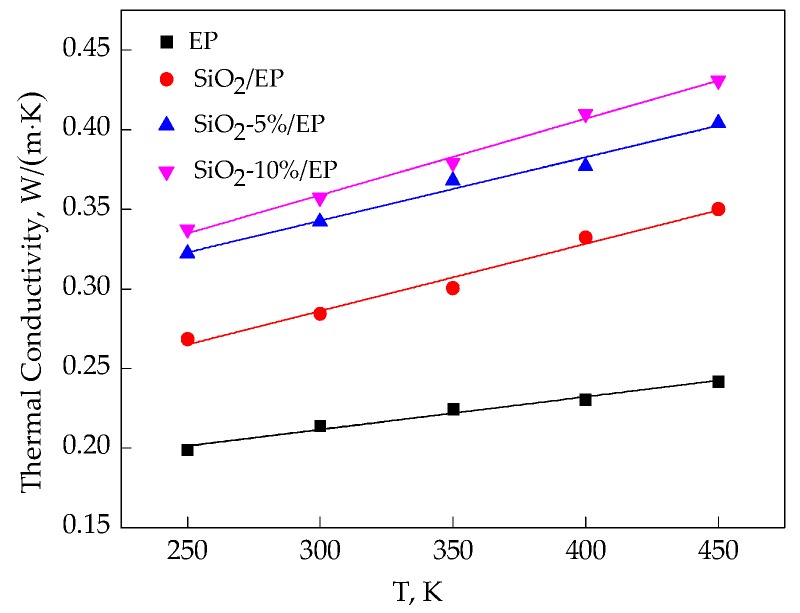
Thermal conductivity fitting curves of four models under different temperatures.

In Table 4, the simulated thermal conductivity of pure epoxy resin is 0.214 W/(m·K) at 300 K, and this value was slightly higher than the experimental result (0.208 W/(m·K)) as reported in Ref. [4]. Defects in the experimental sample may possibly reduce thermal conductivity, while the deviation is within an allowable range. These findings confirmed the feasibility of this method for thermal conductivity calculation. Figure 5 shows the fitting curves of thermal conductivities, and a linear increasing tendency can be seen.

For the silica–epoxy composite model without grafting, the volume fraction of SiO_2_ is 7.2%. Maxwell’s equation (Equation (10)) revealed a calculated thermal conductivity of 0.261 W/(m·K), which is slightly smaller than the simulated result (0.284 W/(m·K)) based on the molecular dynamics method, but this error is also within the allowable range:

(10) $$\lambda_{c}=\frac{2\lambda_{p}+\lambda_{f}+2V_{f}(\lambda_{f}-\lambda_{p})}{2\lambda_{p}+\lambda_{f}-V_{f}(\lambda_{f}-\lambda_{p})}\times \lambda_{p}.$$

We can obtain the following conclusions for the molecular dynamic simulation of thermal conductivity:

1. The four models show a linear increase in thermal conductivity from 300 to 450 K. Incorporation of SiO_2_ nanoparticles can improve the thermal conductivity of epoxy resin. As temperature increases, without grafting procedure, thermal conductivity increases from 33.04% at 300 K to 44.75% at 450 K compared with that of the pure EP model.
2. Grafting silane coupling agents on SiO_2_ nanoparticle surface can considerably enhance the thermal conductivity of composites. A high grafting ratio corresponds to a considerable increase in the thermal conductivity of composite models. At 300 K, the thermal conductivity of SiO_2_–10%/EP model increases by 67.07%, which is higher than that of the pure EP model.

### 3.3. Mechanical Properties

The mechanical properties of the four models at different temperatures were simulated in the Forcite Package of Materials Studio(Accelrys Co., Ltd, San Diego, CA, USA). On the basis of the calculated stiffness matrix, we observed that the pure EP model exhibits anisotropic characteristics in terms of nearly zero values of C_14_, C_15_, C_16_, C_24_, C_25_, C_26_, C_34_, C_35_, C_36_, C_45_, C_46_, and C_56_, whereas the silica–epoxy models are isotropic in terms of the similar values of C_11_, C_22_, C_33_, C_12_, C_13_, C_23_, C_44_, C_55_, and C_66_. Lamé constants can be determined on the basis of the presumed isotropic characteristics of the silica–epoxy model in Equations (11) and (12):

(11) $$\lambda =\frac{1}{3}\left(C_{11}+C_{22}+C_{33}\right)-\frac{2}{3}\left(C_{44}+C_{55}+C_{66}\right),$$

(12) $$\mu =\frac{1}{3}\left(C_{44}+C_{55}+C_{66}\right).$$

**Figure 6:**
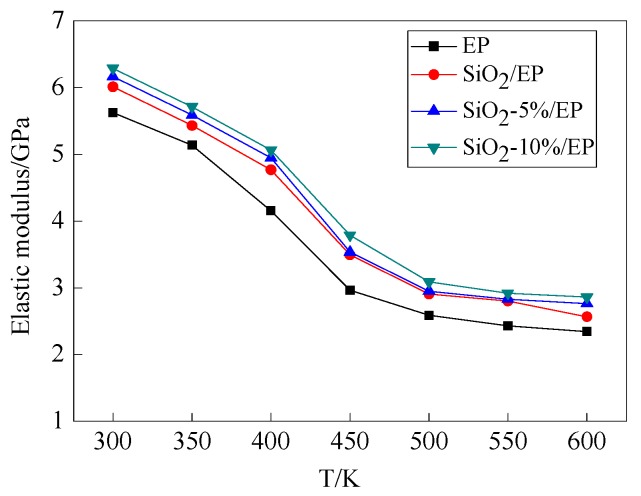
Elastic modulus of four models under different temperatures.

**Figure 7:**
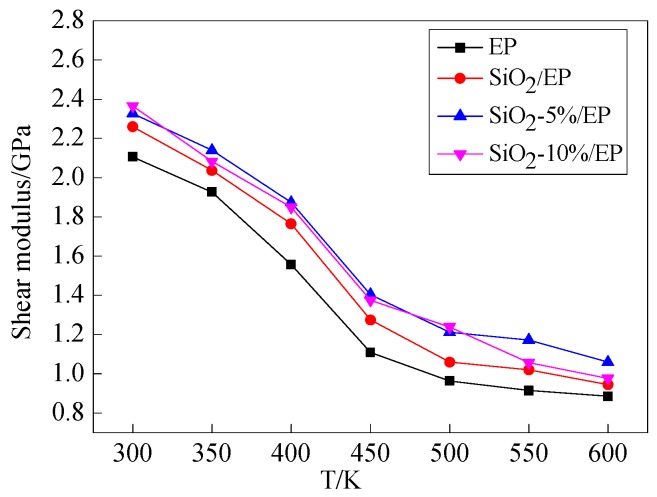
Shear modulus of four models under different temperatures.

According to Equations (4)–(8), the elastic and shear modulus of the four models as a function of temperature are shown in Figure 6 and Figure 7, respectively. Elastic and shear modulus decrease with temperature and reach a nearly balanced value at 500 K. The mechanical properties of the pure EP model rapidly decrease from 350 K, whereas the mechanical properties of the three other models start to decline fast at temperatures exceeding 400 K. These findings confirm that *T*g is improved after SiO_2_ is incorporated. The calculated results are consistent with those reported in previous studies [30,31,32]. The mechanical properties of the composite models with grafting are higher than those of the models without grafting. Likewise, the mechanical properties of the model with a large grafting ratio are enhanced, and these observations indicate that surface modification of SiO_2_ nanoparticles with silane coupling agents can improve the mechanical properties of epoxy resin.

### 3.4. Binding Energy

The combination between polymer hosts and nanoparticles forms new chemical or hydrogen bonds that play important roles in establishing a 3D cross-linked structure. The binding energy of composite models can be calculated using Equation (13) to elucidate the connection mechanism after grafting procedure:

(13) $$\Delta E=E_{\mathrm{total}}-(E_{\mathrm{EP}}+E_{{\mathrm{SiO}}_{2}}),$$

where *E*_total_ represents total energy of the composite models, *E*_EP_ is the total energy of the pure EP model, and *E*_SiO__2_ is the total energy of SiO_2_ nanoparticles. Table 5 shows the final binding energy of the four models at different temperatures.

In Table 5, the binding energy of the silica–epoxy composite models increases as grafting ratio increases, and decreases as temperature decreases. The composite model with 10% grafting ratio exhibits a strong binding energy, which can be attributed to the formation of more hydrogen bonds after grafting. Silane coupling agents not only act as a bridge to connect the EP host and nanoparticles but also help form a tight 3D cross-linked structure with few defects, such as holes, cavities, or cracks [35].

Therefore, grafting silane coupling agent on the surface of SiO_2_ particles can increase the interaction energy between SiO_2_ and EP and thus enhance the mechanical properties of silica–epoxy composites [30,31].

### 3.5. Segment Movement

**Figure 8:**
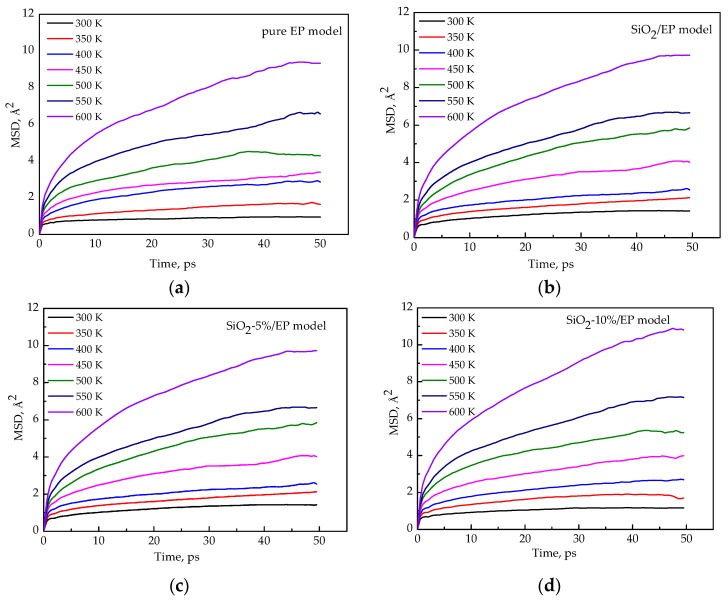
MSD functions for four kinds of models at different temperatures. (**a**) pure EP model; (**b**) SiO_2_/EP model; (**c**) SiO_2_–5%/EP model; (**d**) SiO_2_–10%/EP model.

**Figure 9:**
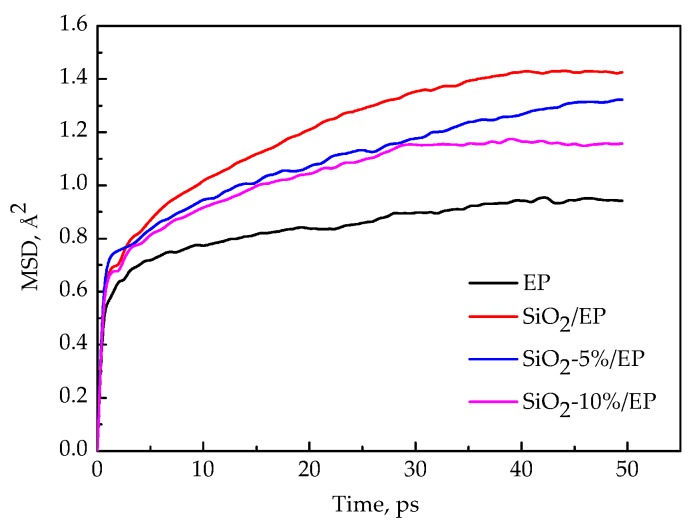
MSD functions for four kinds of models at 300 K.

The plots of the mean square displacement (MSD) as a function of time at different temperatures in the four models (EP, SiO_2_/EP, SiO_2_–5%/EP, and SiO_2_–10%/EP) were analyzed on the basis of the kinematic capacity of the model molecular chain to determine the effects of temperature on the thermodynamic properties of these models. The total simulation time was 50 ps, and one frame was the output for every 0.5 ps. The MSD curves of the four models at different temperatures are shown in Figure 8a–d. Moreover, Figure 9 shows the MSD plots of the four models at 300 K for comparison to reveal the influence of the silane coupling agent KH550 on the segmental mobility of the composite material chain. The following conclusions can be drawn:

1. The MSDs of the four models increase with time until a steady state is reached. At a high temperature, especially above 450 K, the value increases faster, which can be attributed to the resultant larger kinetic energy of the molecular chains when the models attain the glassy state. A strong kinetic energy of a molecular chain can lead to a rapid decline of mechanical properties. These results are consistent with the calculated mechanical properties.
2. Incorporation of SiO_2_ nanoparticles without surface modification into the epoxy matrix may increase the MSD compared with that of the pure epoxy resin model. However, the silica–epoxy model with 10% grafting ratio of the silane coupling agent can reduce the MSD shown in Figure 9. Therefore, surface modification using a silane coupling agent can create a tighter 3D cross-linked structure to enhance the mechanical and thermal properties of composites. A high grafting ratio can also help complete equilibration processes within a short time. Overall, grafting plays a major role in fabricating flexible new composites.

### 3.6. Radial Distribution Function (RDFs)

**Figure 10:**
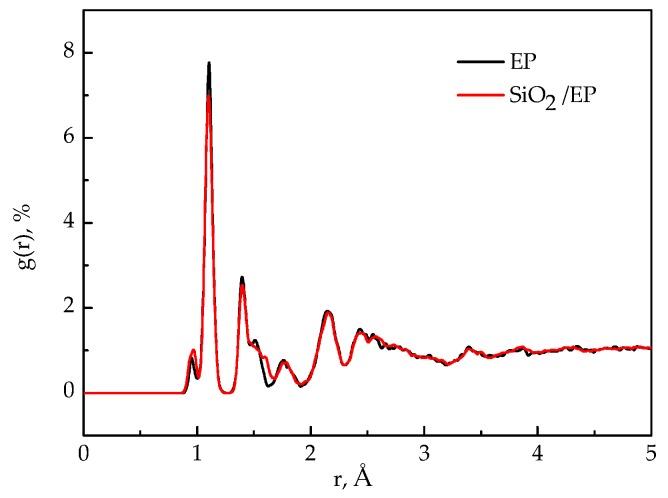
RDF functions before and after incorporating of silica nanoparticles.

**Figure 11:**
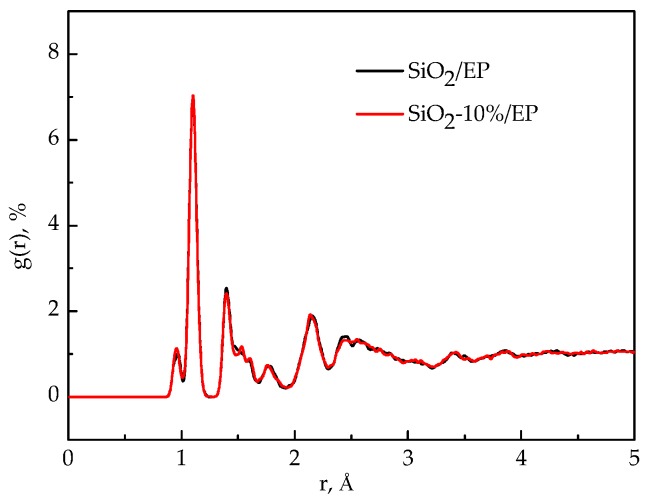
RDF functions before and after grafting modification for silica–epoxy nanocomposites.

RDFs of the models before and after SiO_2_ incorporation were analyzed to examine the structural changes in the entire system after SiO_2_ incorporation and surface modification. Figure 10 shows the RDFs of the pure epoxy resin model and the silica–epoxy composite model without grafting. The peak representing the hydroxyl at 0.96 Å became stronger than that of the pure epoxy resin model, and this observation could account for the increased number of –OH on the surface of SiO_2_ particles. By contrast, the RDF plot of the silica–epoxy composite model with 10% grafting ratio closely resembles the silica–epoxy composite model without grafting. This finding indicated that both models probably yield the same density, as shown in Figure 11. The peaks at 0.96 and 1.61 Å slightly increased, and this phenomenon was attributed to the hydrogen bond interaction produced between the silicon–oxygen bond and hydroxyls.

### 3.7. Free Volume Fractions

**Figure 12:**
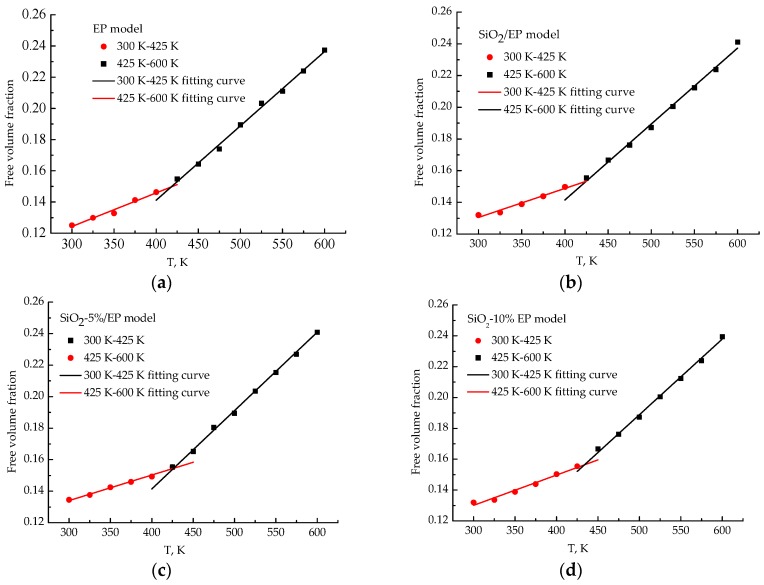
Free volume fractions of four models at different temperatures. (**a**) pure EP model; (**b**) SiO_2_/EP model; (**c**) SiO_2_–5%/EP model; (**d**) SiO_2_–10%/EP model.

Free volume fractions act as the main parameter that indicates and predicts the mechanical property of composites. When composites remain in a glassy state, a relatively small free volume of a system can hinder the movement of molecular segments during a potential deformation process and improves the elastic modulus of the system. Figure 12 shows the free volume fraction of the four models of the cross-linked system as a function of temperature. The free volume fraction increases with temperature and exhibits a large increment at high temperatures. The free volume correspondingly increases because the space required for the movement of molecular chains gradually expands when temperature increases. Large free volume fractions may lead to the decrease of the elastic modulus of a system. Therefore, the analyzed free volume fraction matches well with the results of segmental movement. By contrast, the four respective temperatures at the intersection point in the four models are 417, 424, 426, and 431 K, which can also represent *T*g of the four models. A consistent finding is obtained after comparing the predicted *T*g from the free volume fractions with the calculated *T*g based on specific volume. If the large free volume fractions of the composite models cannot be offset by improving the wettability between particles and polymer hosts, then the mechanical and thermal properties ought to decline. The analyzed free volume fractions confirmed that surface modification is necessary.

## 4. Conclusions

In this study, the mechanical and thermal properties of silica–epoxy composite models were investigated through molecular dynamics simulation. Our results confirmed that surface modification by grafting a silane coupling agent onto a nanoparticle surface is an efficient method to improve the mechanical and thermal properties of composites. The following findings can be obtained through simulation calculation:

1. Incorporating SiO_2_ nanoparticles into a polymer host can effectively and proportionally improve *T*g, mechanical properties, and thermal conductivity. The surface modification of SiO_2_ nanoparticles with silane coupling agent KH550 can further enhance the mechanical and thermal properties of composites. The composite model is further improved when the grafting rate reaches 10%. Compared with the pure epoxy resin model, *T*g of the model of SiO_2_–10%/EP increased by 7%. Although the mechanical properties of silica–epoxy composite models likely decline with temperature, the mechanical properties of the silica–epoxy model were also enhanced after incorporating. In the range of 250 to 450 K, thermal conductivity linearly increased. At 300 K, the thermal conductivity of the SiO_2_–10%/EP model increased by up to 67% at 0.357 W/(m·K), and this value was higher than that of the pure epoxy resin model at 0.214 W/(m·K). The binding energy between SiO_2_ and epoxy resin also increased by up to 38%, demonstrating a strong interaction between polymer hosts and particles after grafting.
2. The analysis of mean square displacement, radial distribution function, and free volume fraction further elucidates the improved mechanism. Temperature drastically influences the three values. The silica–epoxy model with 10% grafting ratio of silane coupling agent could create a tight 3D cross-linked structure and maintain a relatively low MSD value at high temperatures. In the radial distribution function analysis, the peak representing the hydroxyl group at 0.96 Å became stronger than that of the pure epoxy resin model, verifying that the amount of –OH on the modified surface of SiO_2_ particles increased. The model of SiO_2_–10%/EP also shows a lower value of free volume fraction than that of the model of SiO_2_/EP. Overall, grafting procedure proves necessary to control and maintain the improved mechanical and thermal properties.

## Figures and Tables

**Table 1:** Temperature dependence of side length of cubic unit cells for four models (units: Å).

Structure models	600 K	500 K	400 K	300 K
EP	31.25	30.90	30.63	30.55
SiO_2_/EP	40.71	40.09	39.61	39.41
SiO_2_–5%/EP	40.96	40.11	39.69	39.33
SiO_2_–10%/EP	40.58	40.04	39.52	39.32

**Table 2:** Temperature dependence of densities of four models (units: g/cm^3^).

Structure models	600 K	500 K	400 K	300 K
EP	1.015	1.081	1.098	1.13
SiO_2_/EP	1.108	1.164	1.207	1.225
SiO_2_–5%/EP	1.094	1.167	1.204	1.237
SiO_2_–10%/EP	1.124	1.17	1.217	1.235

**Table 3:** *T*g (units: K) and CTE (units: 10^−6^/K) of four models.

Structure models	*T*g	*T*g in Ref.	CTE below *T*g (250–350 K)	CTE above *T*g (450–650 K)	CTE below *T*g in Ref.	CTE above *T*g in Ref.
EP	410	462 ± 9 [33]	218	375	90 [41]	188 [41]
SiO_2_/EP	421	456 ± 11 [33] 414 [3]	206	360		
SiO_2_–5%/EP	427		198	321		
SiO_2_–10%/EP	438		192	295		

**Table 4:** Thermal conductivity of four models under different temperatures (units in W/(m·K)).

Temperature	EP	SiO_2_/EP	SiO_2_–5%/EP	SiO_2_–10%/EP
250 K	0.201	0.265	0.323	0.340
300 K	0.214	0.284	0.342	0.357
350 K	0.225	0.301	0.368	0.379
400 K	0.231	0.332	0.377	0.410
450 K	0.242	0.350	0.404	0.431

**Table 5:** Binding energy of silica–epoxy nanocomposites before and after surface modification. (Δ*E* units in (kcal/mol)).

Temperature	SiO_2_/EP	SiO_2_–5%/EP	SiO_2_–10%/EP
300 K	−423	−461	−585
400 K	−347	−414	−531
500 K	−345	−384	−481
